# COVID-Induced Fulminant Myocarditis

**DOI:** 10.7759/cureus.23894

**Published:** 2022-04-06

**Authors:** Miguel A Rodriguez Guerra, Ramona Lappot, Ana P Urena, Timothy Vittorio, Gabriella Roa Gomez

**Affiliations:** 1 Medicine, Montefiore Medical Center Albert Einstein College of Medicine, Bronx, USA; 2 Internal Medicine, Center for Diagnosis, Advanced Medicine and Telemedicine (CEDIMAT), Santo Domingo, DOM; 3 Medicine, Medicina Cardiovascular Asociada, Santo Domingo, DOM; 4 Cardiovascular Disease, BronxCare Health System, Bronx, USA; 5 Pulmonary and Critical Care Medicine, Montefiore Medical Center Albert Einstein College of Medicine, Bronx, USA

**Keywords:** sars-cov-2, fulminant myocarditis, sudden death, myocarditis, coronavirus

## Abstract

Viral-induced myocarditis has different presentations, from being asymptomatic to fatal arrhythmias. It is crucial to recognize and treat this condition early to improve morbidity and mortality.

We report a case of a 56-year-old male who tested positive for severe acute respiratory syndrome coronavirus 2 (SARS-CoV-2) three days ago and presented with syncope. The physical exam was relevant for right eyebrow laceration, tachycardia, and hypotension that responded to intravenous fluid, but two hours later, he had mental status changes, bradycardia, hypotension, and cardiac arrest. His repeated electrocardiogram (ECG) showed diffuse ST-segment elevation. Troponemia was evident in his blood work. Point-of-care ultrasound (POCUS) at the bedside showed dilated cardiomyopathy. Unfortunately, the patient re-arrested and needed advanced cardiovascular life support (ACLS).

The initial assessment of SARS-CoV-2, serial ECGs, and cardiac markers are essential for a prompt approach and therapy in COVID-19-induced myocarditis.

## Introduction

Myocarditis could represent a challenge due to its multiple clinical presentations [1]. This condition could be complicated by left heart failure or arrhythmias leading to poor outcomes [2]. In severe acute respiratory syndrome coronavirus 2 (SARS-CoV-2) infection, myocarditis could present as acute coronary syndrome (ACS) but could also be asymptomatic with ECG and echocardiographic changes and their mechanism. A possible mechanism of this viral myocarditis is based on the myocardial injury due to the elevated immune response, renin-angiotensin-aldosterone system dysregulation, and endothelial injury [3].

A definitive diagnosis of myocarditis would require an endomyocardial biopsy (EMB). However, it is not done in most cases, but the European Society of Cardiology (ESC) created criteria by expert consensus in coordination with the World Health Organization (WHO) [4].

This is a compelling case of an uncommon presentation of SARS-CoV2-induced myocarditis in a patient that arrested twice and unfortunately presented sudden cardiac death after ventricular arrhythmia.

## Case presentation

The patient was a 56-year-old male who Emergency Medical Services (EMS) brought in due to a syncopal episode after trying to sit down and losing consciousness, sustaining a laceration on his right eyebrow. EMS reported that the patient was found to be hypoxic, saturating at 76%. The patient tested positive for coronavirus disease (COVID) in the community less than one week ago. Since then, he had weakness and fatigue and vomited twice. He had a history of hypertension and diabetes and denied a prior history of carditis or cardiomyopathy. The physical exam showed a right eyebrow laceration with bruises in the surrounding area. However, he was tachycardic (107 bpm) and hypotensive with a systolic blood pressure of 93 mmHg and responded to normal saline 1-liter bolus.

**Figure 1:**
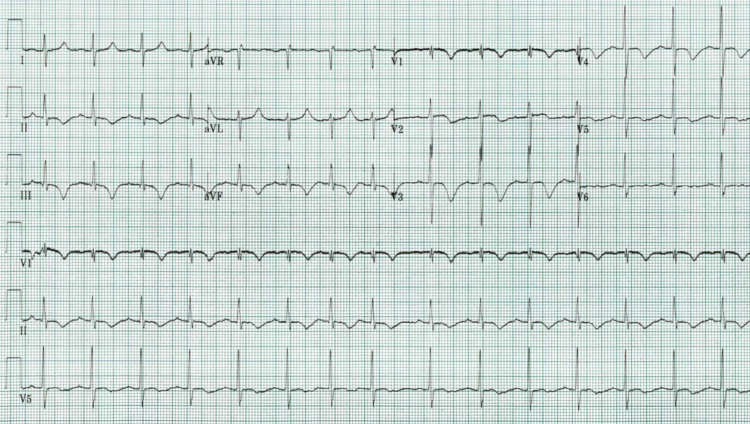
Initial ECG T-wave inversion and prolonged QTc in the inferior and antero-apical leads

His initial ECG showed T-wave inversion in the inferior and antero-apical leads (II, III, V1, V2, V3, V4) as well as prolonged QTc (540 msec) (Figure 1).

His blood work was normal except for anion gap acidosis, lactic acidosis, acute kidney injury (AKI) on chronic kidney disease (CKD), elevated C-reactive protein (CRP), troponemia, and COVID polymerase chain reaction (PCR) positive (Table 1). CT head was negative for acute findings.

**Table 1:** Lab results HCO: bicarbonate; GFR: glomerular filtration rate; CRP: C-reactive protein; SARS-CoV-2: severe acute respiratory syndrome coronavirus 2

	Value	Reference
pH	7.274	7.310 - 7.410 pH units
Lactate	4.6	0.5 - 2.0 mmol/L
HCO	18	22.0 - 30.0 mmol/L
Anion gap	15	8.00 - 12.00 mmol/L
Creatinine	1.60	0.80 - 1.50 mg/dl
GFR	45.00	> 90
CRP	25.87	.01 - 5.0 mg/L
Troponin quantitative	0.30	00 - 0.04 ng/mL
D-dimer high sensitive	13.38	0.19 - 0.50 mg/L FEU
SARS-CoV-2	Positive	-

**Figure 2:**
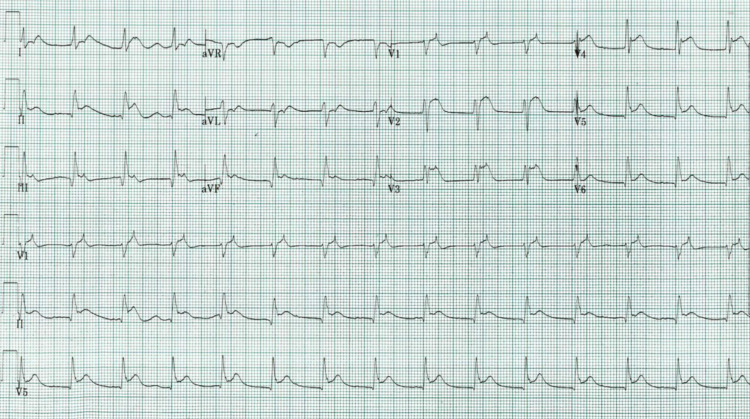
Second ECG New ST-segment elevation

Then, three hours after his presentation, the patient became confused, vomited, turned bradycardic, hypotensive, with agonal breathing, and then became pulseless. Endotracheal intubation was done, and return of spontaneous circulation (ROSC) was obtained after 10 mins of ACLS. The second ECG, two hours after the initial, showed diffused ST-segment elevations (Figure 2); the ST-segment elevation myocardial infarction (STEMI) code was called.

**Figure 3:**
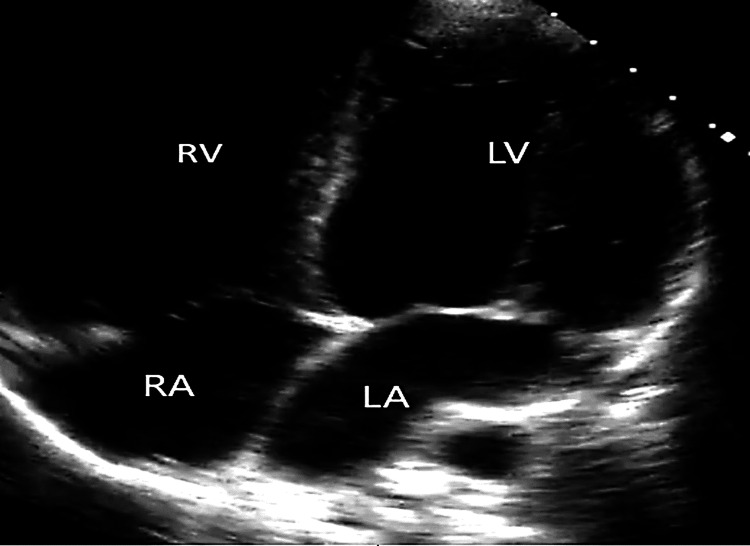
POCUS showing a dilated cardiomyopathy evident in the apical four-chamber view RV: right ventricle; LV: left ventricle; RA: right atrium; LA: left atrium

Point-of-care ultrasound (POCUS) at the bedside showed dilated cardiomyopathy in the four-chamber view (Figure 3).

The cardiology team reported that coronary syndrome was unlikely because this patient had not had this viral infection in the past and was currently in the acute phase. They concluded that the patient was most probably coursing with COVID-induced myocarditis due to the absence of chest pain, involvement of right and left coronary abnormalities in the ECG, and current coronavirus infection.

Besides, the ECG could be concerning for ACS (possibly Wellen's syndrome), right coronary artery (RCA) territory, which would not be part of Wellen's criteria. The acute viral infection with the ECG changes in the setting of elevated inflammatory markers and the absence of chest pain would favor the diagnosis of myocarditis.

The patient sustained another cardiac arrest after a ventricular tachycardia was noticed and had 30 mins of unsuccessful ACLS.

## Discussion

Myocarditis is a cardiac muscle inflammatory reaction at the level of the myocardium that multiple causes could trigger (infectious or non-infectious) [5]. His inflammatory process led to myocardial dysfunction and dilated cardiomyopathy [6]. The Lombardy registry showed that 26.6% of patients with suspected myocarditis had a low ejection fraction, ventricular arrhythmia, or low cardiac output syndrome; these patients had worse outcomes [7].

Unfortunately, patients with myocarditis cannot typically be described with specific symptoms; a certain amount of patients can have a subclinical course [8]. However, commonly, its manifestations are not specific, for example, chest pain, new-onset heart failure, cardiac chamber or chambers dilation, fatigue, new arrhythmias, and the worst-case scenario is sudden death [9].

A myocardial biopsy is controversial; not all patients are candidates and could represent a significant complication while non-invasive methods like the electrocardiogram (ECG), echocardiogram, and cardiac MRI could be used [10-11]. The ECG would show QRS amplitude changes, PR depression, non-specific ST changes, or depression or elevation. The echocardiogram typically shows new chamber (one or more) dilation or low ejection fraction, and the MRI commonly shows contractile dysfunction, myocardial edema, and reversible injury [12-13].

Thirty-six percent (36%) of patients with myocarditis diagnosed by biopsy had a history of recent upper respiratory infection or enteritis. Some viruses are known to be related to myocardial injury, including parvovirus and coxsackie A. The reason why a viral infection with tachycardia that is higher than expected due to the fever should be investigated [6,14].

COVID-induced myocarditis has been reported, but it is uncommon. Its mechanism is thought to be via the hyperinflammatory reaction as a consequence of the cytokine storms; it has been related to poor outcomes [15]. Hoffman et al. exposed the interaction of the SARS-CoV receptor angiotensin-converting enzyme 2 (ACE2) and the serine protease TMPRSS2 for the S protein priming while Hassan et al. exposed the cellular injury in the pericytes and endothelium [16]. Due to potential complications, the non-invasive method is preferred over EMB, and the clinicians base their clinical findings [17]. The European Society of Cardiology (ESC)/WHO stated specific criteria for “clinically suspected myocarditis,” establishing the need for at least one clinical and one diagnostic criterion (Table 2) [18].

**Table 2:** Myocarditis clinical criteria * I to III degree atrioventricular block, or bundle branch block, ST/T wave change (ST elevation or non-ST elevation, T wave inversion), sinus arrest, ventricular tachycardia or fibrillation and asystole, atrial fibrillation, reduced R wave height, intraventricular conduction delay (widened QRS complex), abnormal Q waves, low voltage, frequent premature beats, supraventricular tachycardia

Clinical Presentation	Diagnostic Criteria
Acute chest pain pericardic or pseudo-ischemic	Newly abnormal ECG and/or Holter and or stress test*
New-onset dyspnea or fatigue with or without heart failure sign	Elevated troponin
Subacute or chronic dyspnea or fatigue with or without heart failure sign	Functional or structural abnormality evident on imaging
Palpitation, and/or unexplained arrhythmia and/or syncope and/or aborted sudden cardiac death	Evidence on tissue sample
Unexplained cardiogenic shock	

Although the application of the consensus criteria is recommended, the patient should be evaluated for a possible EMB, especially in a young patient without significant comorbidities because its results could help identify the condition, its etiology, and possibly guide therapy [19-20].

Our patient is an exceptional SARS-CoV-positive case who had new-onset dyspnea, tachycardia, cardiogenic shock, new ECG changes, sudden cardiac death, aborted cardiac death, new-onset dilated cardiomyopathy, and then a non-aborted cardiac death.

## Conclusions

COVID-induced myocarditis is uncommon but the initial assessment of these patients is crucial due to the high mortality associated with myocarditis. The diagnosis of this condition is multifactorial because of the multiple heterogeneities of its presentation. Its manifestation could represent a challenge for the clinician, commonly basing its diagnosis on ruling out other diseases, including acute coronary syndrome. In the absence of chest pain, clinical suspicions are more difficult to make. However, if the cardiac markers are abnormal, an expedited cardiac image would be essential to identify a possible cardiac complication. The outcome of this condition could be associated with the inflammatory reaction caused by the cytokine storm in this viral infection. Fulminant presentation is not frequent, but serial ECGs and cardiac markers are essential for a prompt approach and therapy.

## References

[1] Schultheiss HP, Baumeier C, Aleshcheva G, Bock CT, Escher F (2021). Viral myocarditis—from pathophysiology to treatment. J Clin Med.

[2] Tschöpe C, Ammirati E, Bozkurt B (2021). Myocarditis and inflammatory cardiomyopathy: current evidence and future directions. Nat Rev Cardiol.

[3] Ozieranski K, Tyminska A, Jonik S (2021). Clinically suspected myocarditis in the course of severe acute respiratory syndrome novel coronavirus-2 infection: fact or fiction?. J Card Fail.

[4] Caforio A L, Pankuweit S, Arbustini E et, al. al. (2013). Current state of knowledge on aetiology, diagnosis, management, and therapy of myocarditis: a position statement of the European Society of Cardiology Working Group on Myocardial and Pericardial Diseases. Eur Heart J.

[5] Cooper LT Jr (2009). Myocarditis. N Engl J Med.

[6] Kearney MT, Cotton JM, Richardson PJ, Shah AM (2001). Viral myocarditis and dilated cardiomyopathy: mechanisms, manifestations, and management. Postgrad Med J.

[7] Ammirati E, Cipriani M, Moro C (2018). Clinical presentation and outcome in a contemporary cohort of patients with acute myocarditis. Multicenter Lombardy Registry. Circulation.

[8] Fung G, Luo H, Qiu Y, Yang D, McManus B (2016). Myocarditis. Circ Res.

[9] Lampejo T, Durkin SM, Bhatt N, Guttmann O (2021). Acute myocarditis: aetiology, diagnosis and management. Clin Med (Lond).

[10] Liguori C, Farina D, Vaccher F, Ferrandino G, Bellini D, Carbone I (2020). Myocarditis: imaging up to date. Radiol Med.

[11] Bière L, Piriou N, Ernande L, Rouzet F, Lairez O (2019). Imaging of myocarditis and inflammatory cardiomyopathies. Arch Cardiovasc Dis.

[12] Friedrich MG, Marcotte F (2013). Cardiac magnetic resonance assessment of myocarditis. Circ Cardiovasc Imaging.

[13] Doyen D, Moceri P, Ducreux D, Dellamonica J (2020). Myocarditis in a patient with COVID-19: a cause of raised troponin and ECG changes. Lancet.

[14] Al-Akchar M, Kiel J (2022). Acute Myocarditis. https://www.ncbi.nlm.nih.gov/books/NBK441847/.

[15] Bami K, Haddad T, Dick A, Dennie C, Dwivedi G (2016). Noninvasive imaging in acute myocarditis. Curr Opin Cardiol.

[16] Abou Hassan OK, Sheng CC, Wang TK, Cremer PC (2021). SARS-CoV-2 myocarditis: insights into incidence, prognosis, and therapeutic implications. Curr Cardiol Rep.

[17] Jeserich M, Konstantinides S, Pavlik G, Bode C, Geibel A (2009). Non-invasive imaging in the diagnosis of acute viral myocarditis. Clin Res Cardiol.

[18] Ponikowski P, Voors AA, Anker SD (2016). 2016 ESC guidelines for the diagnosis and treatment of acute and chronic heart failure. The Task Force for the diagnosis and treatment of acute and chronic heart failure of the European Society of Cardiology (ESC). Developed with the special contribution of the Heart Failure Association (HFA) of the ESC. Eur J Heart Fail.

[19] Seferović PM, Tsutsui H, McNamara DM (2021). Heart Failure Association of the ESC, Heart Failure Society of America and Japanese Heart Failure Society position statement on endomyocardial biopsy. Eur J Heart Fail.

[20] Kawakami R, Sakamoto A, Kawai K (2021). Pathological evidence for SARS-CoV-2 as a cause of myocarditis: JACC review topic of the week. J Am Coll Cardiol.

